# The Integration of ANN and FEA and Its Application to Property Prediction of Dual-Performance Turbine Disks

**DOI:** 10.3390/ma17133045

**Published:** 2024-06-21

**Authors:** Yanqing Li, Ziming Zhang, Junyi Cheng, Zhaofeng Liu, Chao Yin, Chao Wang, Jianzheng Guo

**Affiliations:** 1 Wedge Central South Research Institute Co., Ltd., Shenzhen 518045, China; liyanqinglyq@126.com (Y.L.);; 2 Department of Mathematics, The University of British Columbia, Vancouver, BC V6T1Z4, Canada; 3 State Key Laboratory of Powder Metallurgy, Central South University, Changsha 410083, China; 4 Shenzhen Wedge Aviation Technology Co., Ltd., Shenzhen 518000, China

**Keywords:** artificial neural network, dual microstructure heat treatment, finite element, Ni based super alloy, turbine disks

## Abstract

Regulating the microstructure of powder metallurgy (P/M) nickel-based superalloys to achieve superior mechanical properties through heat treatment is a prevalent method in turbine disk design. However, in the case of dual-performance turbine disks, the complexity and non-uniformity of the heat treatment process present substantial challenges. The prediction of yield strength is typically derived from the analysis of microstructures under various heat treatment regimes. This method is time-consuming, expensive, and the accuracy often depends on the precision of microstructural characterization. This study successfully employed a coupled method of Artificial Neural Network (ANN) and finite element analysis (FEA) to reveal the relationship between the heat treatment process and yield strength. The coupled method accurately predicted the location specified and temperature-dependent yield strength based on the heat treatment parameters such as holding temperatures and cooling rates. The root mean square error (RMSE) and mean absolute percentage deviation (MAPD) for the training set are 50.37 and 3.77, respectively, while, for the testing set, they are 50.13 and 3.71, respectively. Furthermore, an integrated model of FEA and ANN is established using a Abaqus user subroutine. The integrated model can predict the yield strength based on temperature calculation results and automatically update material properties of the FEA model during the loading process simulation. This allows for an accurate calculation of the stress–strain state of the turbine disk during actual working conditions, aiding in locating areas of stress concentration, plastic deformation, and other critical regions, and provides a novel reliable reference for the rapid design of the turbine disk.

## 1. Introduction

Nickel-based superalloys exhibit exceptional high-temperature strength, fatigue performance, and creep resistance, making them indispensable for critical high-temperature components in aeroengines, such as turbine disks and blades [1,2]. Advanced powder turbine disks are typically formed through Hot Isostatic Pressing (HIP), Heat Extrusion (HEX), and Isothermal Forging (IF), followed by heat treatment to tailor the microstructure [3]. The optimization of heat treatment processes to achieve the desired microstructure, thus ensuring high-performance turbine disks, has become a hot research topic.

The exceptional high-temperature mechanical properties of nickel-based P/M superalloys are mainly attributed to the γ′ phase precipitation strengthening [4,5,6]. The ordered L12 structural γ′ phase is embedded in and atomically coherent with the face-centered cubic γ phase. Therefore, the mechanical properties of the P/M superalloys will be influenced by the morphology, size, volume fraction, and distribution of the γ′ phase. Most characteristics of the γ′ phase can be adjusted by subsolvus or supersolvus heat treatment processes to achieve an optimal configuration. In addition, grain size also significantly affects the mechanical properties of polycrystalline alloys [7]. A fine grain structure of superalloys proves advantageous in resisting deformation at low temperatures, while also hindering the development of grain facet fatigue cracks [8]. On the contrary, superalloys with coarse grain structures exhibit superior resistance to both creep and crack growth processes, especially at elevated temperatures. In the traditional manufacturing process of the turbine disk, the microstructure and mechanical property distribution across the disk after heat treatment are approximately uniform. However, during the operation conditions of the aeroengine turbine disk, the disk hub endures a combination of low temperature and high stress, while the rim is exposed to higher temperatures and lower stress. Therefore, the turbine disk should be fabricated according to the performance requirements of specific positions on the turbine disk.

The Dual Microstructure Heat Treatment (DMHT) method was developed to produce dual-performance turbine disks [9,10]. This technology utilizes specially designed heat treatment insulation and furnaces to heat treat the turbine disk. It creates a temperature gradient in the hub and rim areas of the disk, resulting in differentiated microstructures in different areas. To address the high-stress conditions in the disk hub and the low-stress conditions in the disk rim, the utilization of the DMHT method as a heat treatment approach for the disk can effectively meet the performance requirements for the turbine disk.

The DMHT requires a well-designed insulation and heat treatment parameters to meet the performance requirements for the dual-performance turbine disk. At present, many scholars have conducted studies on predicting the mechanical performance of dual-performance turbine disks [11,12,13,14,15]. The strengthening mechanisms, including weak and strong dislocation coupling and anti-phase boundary, have been widely used to describe the relationship between microstructure and mechanical properties. However, when investigating the relationship between microstructure and mechanical properties through alloy strengthening mechanisms, extensive characterization of the alloy is typically required. Furthermore, the accuracy of predictions heavily depends on the precision of microstructural characterization, whereas Machine Learning (ML) is an effective alternative to overcome these issues. ML is the method that focuses on creating algorithms capable of learning from, predicting, or categorizing data which has been widely employed in the field of materials science [16,17,18,19]. For instance, ML methodologies have been employed to characterize material behaviors such as deformation [20], recrystallization [18], work-hardening [21], and even the compositional design of new alloys [22]. However, predicting the performance of DMHT turbine disks using machine learning is still challenging, and there have been no previous reports on this topic based on the authors’ knowledge. In this study, we successfully employed a coupling of machine learning and finite element simulations to accurately predict DMHT disk performance. We also developed an integrated model using a user-defined subroutine to combine ANN and FEA. This model allows the simulation of the turbine disk behavior under loading conditions, where the yield strength is predicted based on temperature calculations, and then the predicted yield strength is automatically updated into the yield strength of the FEA model for subsequent stress–strain analyses. This approach effectively reduces testing costs and offers novel insights for the rapid design of turbine disk in the future.

## 2. Fundamentals of FEA and ANN Coupled Method

This study employs a coupled approach of the FEA model and ANN model to predict the yield strength of DMHT disks and establish an integrated model of FEA and ANN for subsequent simulation of turbine disks.

**Figure 1:**
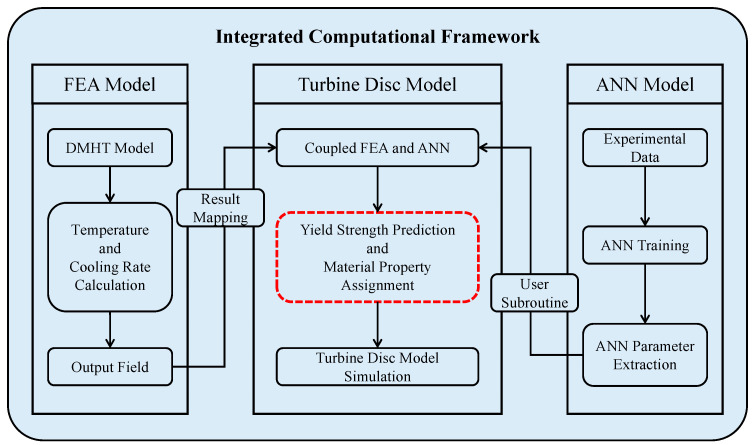
Integrated computational framework.

Compared to traditional methods that use alloy strengthening mechanisms to predict mechanical properties, this approach bypasses the complex and cumbersome process of building “process-microstructure-property” models. Instead, it utilizes the machine learning technique to establish direct mapping relationships between heat treatment parameters and mechanical properties. By embedding this mapping relationship into the turbine disk FEA model and using temperature histories of the DMHT simulation as the input data, the predicted property distribution of turbine disks can be achieved. Moreover, in the operating condition process simulations, the yield strength attribute of the disk FEA model can be automatically updated based on the temperature distribution during the operating process and then used for subsequent stress and strain simulations. The integrated computational framework is shown in Figure 1.

### 2.1. ANN Model

ANN with Back Propagation (BP) algorithms has become a vital tool in the study of supper alloy. By modeling complex, nonlinear relationships among processing conditions, microstructure, and mechanical properties, ANNs trained with BP can predict material behavior with significant accuracy. This computational approach allows researchers to circumvent traditional physical and empirical models, facilitating a more efficient design and the optimization of materials and manufacturing processes.

The input data are passed through the network from the input layer to the output layer, computing the activation at each node using the weights and activation function. If we represent activation with a and the activation function with f, the activation at each layer l can be calculated as:

(1) $$a^{\left(l\right)}=f(W^{\left(l\right)}a^{\left(l-1\right)}+b^{\left(l\right)})$$

where $W^{\left(l\right)}$ and $b^{\left(l\right)}$ are the weights and biases for the $l_{th}$ layer, respectively.

The loss function L, such as the least absolute deviations in this study, is used to measure the difference between the network’s output and the target values:

(2) $$L=\sum_{k}\left|y_{k}-t_{k}\right|$$

where k is the number of values, $y_{k}$ is the predicted value, and $t_{k}$ is the target value.

By propagating the gradient of the loss with respect to each weight back through the network, the weights are updated. The gradient at the output layer is:

(3) $$\delta^{\left(l\right)}=sign\left(y-t\right)\cdot f^{\prime }\left(z^{\left(l\right)}\right)$$

where $z^{\left(l\right)}$ is the weighted sum of the $l_{th}$ layer and sign(x) is the sign function, returning +1 if x>0, −1 if x<0, and 0 if x=0.

The gradient at the hidden layers is:

(4) $$\delta^{\left(l\right)}=\left(W^{(l+1)T}\delta^{l+1}\right)\cdot f^{\prime }\left(z^{\left(l\right)}\right)$$

Update weights and biases:

(5) $$W^{\left(l\right)}=W^{\left(l\right)}-\alpha \delta^{\left(l\right)}{\left(a^{\left(l-1\right)}\right)}^{T}$$

where T is the matrix transpose.

(6) $$b^{\left(l\right)}=b^{\left(l\right)}-\alpha \delta^{\left(l\right)}$$

Here, α is the learning rate, controlling the step size of the updates. $f^{\prime }$ represents the derivative of the activation function.

**Figure 2:**
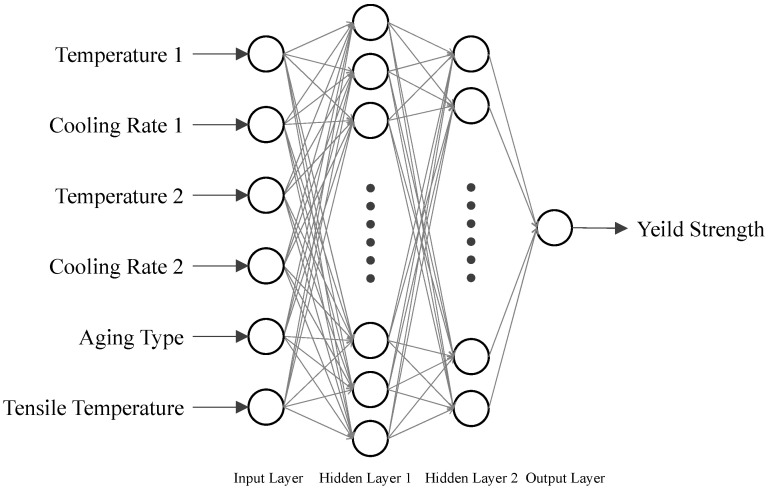
Architecture of an ANN model with two hidden layers.

In the exploration of the heat treatment process, the relationship between the microstructure of the material and the heat treatment parameters was identified. The microstructure significantly influences the yield strength of the material. Accordingly, an ANN model was constructed with two hidden layers, utilizing heat treatment parameters as the input parameters and yield strength as the output, as illustrated in Figure 2. Given that this study involves considering a possible three-step heat treatment process, the parameters for the first step are denoted as temperature-1 and cooling rate-1, the second step parameters are labeled as temperature-2 and cooling rate-2, and the third step parameter is referred to as aging type. The tensile temperature represents the experimental temperature at which the predicted yield strength is evaluated.

Normalization is a pivotal step in the data processing phase. This procedure mitigates the influence of differing dimensions, enhances the precision of the model, and simplifies the underlying data structure. Prior to model training, all input and output data have been normalized to fall within the range of [0,1], a process which can be mathematically expressed as follows:

(7) $$x^{\prime }=\frac{x-x_{min}}{x_{max}-x_{min}}$$

As illustrated in the given expression, the normalized data are denoted by $x^{\prime }$, while x, $x_{min},$ and $x_{max}$ represent the original data, the minimum value, and the maximum value of the original data, respectively. Each dimension is normalized separately within its specific range.

Adding dropout layers in an ANN model enhances its robustness and generalization performance. Dropout is a regularization technique that randomly deactivates a portion of neuron nodes during training to reduce the model’s tendency to overfit. This means that, in each training iteration, only a subset of neurons participates in forward and backward propagation, making the model more capable of generalizing.

During forward propagation, for each training sample, the dropout layer randomly deactivates a fraction of neuron nodes with a probability. This can be represented as:

(8) $$r_{i}\;\sim \;Bernoulli(p)$$

Here, $r_{i}$ represents a binary random variable indicating whether the $i_{th}$ neuron remains active, and p represents the probability of keeping a neuron active.

During backward propagation, the gradients of the deactivated neuron nodes are ignored, and only the gradients of the active neurons are considered for parameter updates.

### 2.2. FEA Model of DMHT

**Figure 3:**
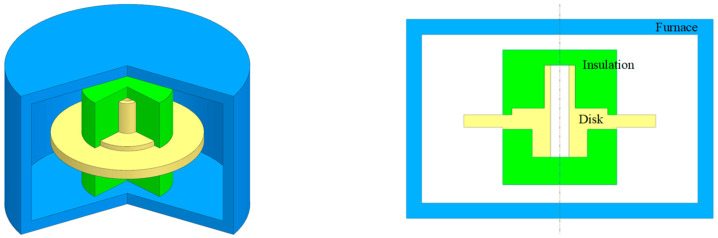
Schematic of dual-performance heat treatment components assembly.

To analyze the impact of strengthening phases on material mechanical properties during the heat treatment process, it is essential to accurately measure the temperature distribution within the turbine disk during heat treatment. Traditional methods using thermocouples are unable to provide comprehensive and precise temperature measurements across the entire turbine disk. To address this challenge, this study employs finite element simulations to model the heat treatment process of the turbine disk and calculates temperature distribution and trends throughout the heat treatment process, providing valuable data for subsequent analysis [23]. The heat treatment model for the turbine disk is illustrated in Figure 3, including the turbine disk, insulation, and vacuum furnace. The DMHT process was analyzed in ABAQUS 2021. The temperature of the heat treatment and the cooling rates, both of which would be input to the ANN model, were calculated by Python scripting in the postprocess.

### 2.3. The Integrated Model of FEA and ANN and the Analysis of Turbo Disk

To predict the yield strength of turbine disks and utilize the predicted yield strength as material properties of the FEA model for subsequent calculations, this study proposes an integrated method of FEA and ANN. The pretrained ANN model, along with the weights and biases corresponding to each neuron, was transferred into the ABAQUS 2021 user-defined subroutine USDFLD. The yield strength was linked to a user-defined field. The input data, such as HT temperatures and cooling rates, were initialized in the other user-defined fields that were mapped from the DMHT simulation results. Finally, an actual working condition analysis was performed on the turbine disk according to the aeroengine ground test conditions.

## 3. Materials and Experiments

In this study, a full-size dual-performance turbine disk made of the P/M superalloy FGH4113A (WZ-A3) was successfully fabricated. The nominal chemical composition is shown in Table 1. This alloy is a precipitation-strengthened nickel-based powder metallurgy superalloy, with the γ′ phase serving as the principal strengthening phase.

**Figure 4:**
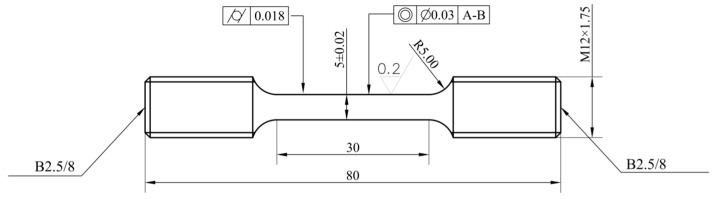
Sketch of tensile specimen.

The preparation process of the disk was vacuum argon atomization → hot isostatic pressing → hot extrusion → isothermal forging → dual-performance heat treatment. The heat treatment comprised three distinct steps. First, subsolvus heat treatment at 1120 °C was applied to ensure and improve the stability of the initial structure. Secondly, the disk underwent supersolvus heat treatment at 1200 °C with thermal insulation covering the hub to create a temperature gradient along its radial axis, promoting grain coarsening at the rim while maintaining the fine grain structure at the hub. Finally, aging heat treatment at 815 °C for 8 h improved the mechanical properties of the disk. A series of tensile tests were carried out on samples obtained from different regions of the full-size disk to ensure an accurate representation of the material’s performance. Figure 4 is the sketch of the tensile specimen.

**Figure 5:**
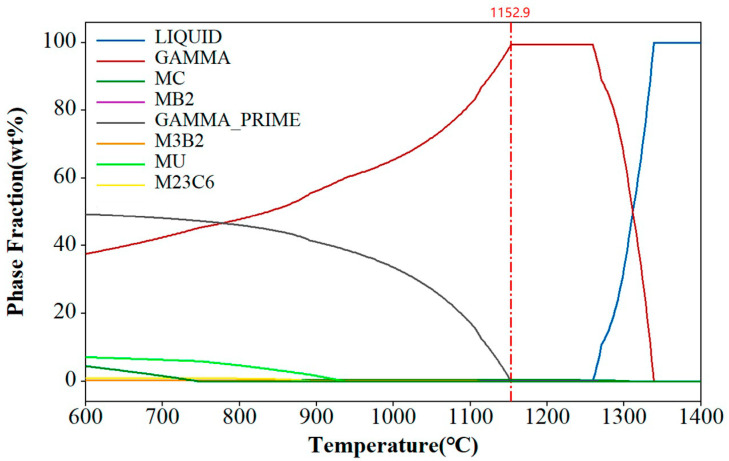
CALPHAD result for the FGH4113A alloy.

The γ′ phase solvus temperature is dependent on the alloy’s chemical composition and can be calculated using the CALPHAD method. For the FGH4113A alloy, the solvus temperature for the γ′ phase was estimated at 1152.9 °C, as shown in Figure 5. When the heat treatment temperature reached or exceeded the solvus temperature of the γ′ phase, also known as supersolvus temperature, the γ′ phase dissolved and reprecipitated during cooling. Controlling the cooling rate during this process allows for the adjustment of the γ′ phase morphology and size distribution. Rapid quenching leads to the formation of unimodal fine γ′ phases, while slow cooling results in the development of a multimodal γ′ phase distribution [24,25,26,27].

The subsolvus heat treatment was conducted at a temperature below the γ′ phase’s solvus temperature. In this case, part of the γ′ phase remains in the alloy, particularly the large primary γ′ phase with a high body surface area ratio. Since the higher diffusion rate of elements at grain boundaries and the coarsening of the γ′ phase following the Oswald–Ripening mechanism, there are larger γ′ phases at grain boundaries. They also play a role as obstacles to limiting grain growth at relative high temperatures according to thr Zener pinning effect. However, during the subsolvus heat treatment, the majority of intergranular γ′ phase dissolved. This enables the combination of fine grain structure and fine precipitates, resulting in superior performance for tensile strength and fatigue resistance up to a medium temperature [28].

## 4. Results

### 4.1. Tensile Experiment Results

**Figure 6:**
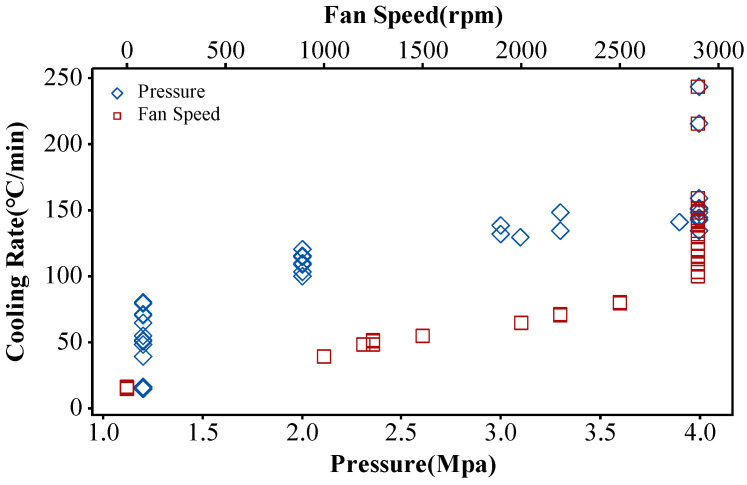
The relationship between cooling rate and pressure as well as fan speed in a vacuum furnace.

In this study, the ANN training dataset was constructed based on the tensile experimental results of a medium-sized experimental disk fabricated during our research process. As yield strength is primarily determined by the microstructure of the alloy after heat treatment, the heat treatment parameters were included as feature parameters in the ANN model. The thermal treatment temperature in the ANN model is defined as the temperature at the beginning of each thermal treatment cooling process, and the cooling rate is defined as the average cooling rate from the thermal treatment temperature to 800 °C. Only two aging modes were used in the heat treatment process: one involved an 815 °C hold for 8 hours, and the other involved a 760 °C hold for 16 hours. Therefore, in the ANN model, the aging types were represented by 0 and 1. These tensile specimens were subjected to a three-step heat treatment process in a vacuum furnace to simulate the heat treatment process of the full-size disk. The gas quenching pressure and fan speed were adjusted to simulate the cooling rate at different disk regions shown in Figure 6.

### 4.2. ANN Model Training Results

In the training of the ANN model in this study, the activation function was defined as a Rectified Linear Unit (ReLU), and the optimization was carried out using the Adaptive Moment Estimation (Adam) algorithm. Appropriate hyperparameters were carefully chosen for this ANN model, with a learning rate of 0.005 and a total of 2000 epochs. Additionally, prior to model training, all input and output data were normalized within a [0,1] range. Dropout layers were also incorporated between the hidden layers to mitigate overfitting on the training data and enhance the model’s generalization performance.

To evaluate the performance of the BP-ANN model, *RMSE* metrics and the *MADP* indicators were applied. *RMSE* and *MADP* quantify the difference between the predicted and observed values, offering insights into the model’s accuracy. Lower *RMSE* and *MADP* values signify better alignment between predictions and actual observations. Together, these metrics provide a well-rounded assessment of the model’s ability to predict the outcomes accurately. The formula for calculating *RMSE* and *MADP* are given by:

(9) $$RMSE=\sqrt{\frac{1}{n}\sum_{i=1}^{n}{\left(y_{i}-{\hat{y}}_{i}\right)}^{2}}$$

(10) $$MADP=\frac{1}{n}\sum_{i=1}^{n}\frac{\left(y_{i}-{\hat{y}}_{i}\right)}{{\hat{y}}_{i}}$$

Here, $y_{i}$ is the actual value, ${\hat{y}}_{i}$ is the value predicted by the model, $\bar{y}$ is the mean of the actual values, and n represents the total amount of data.

**Figure 7:**
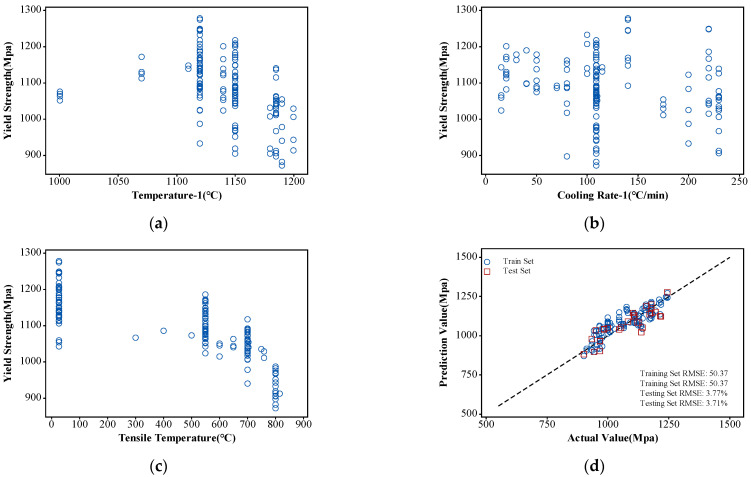
The partial characteristic parameters and tensile strength of the training dataset and ANN model training results: (**a**) distribution of yield strength with respect to temperature-1; (**b**) distribution of yield strength with respect to cooling rate-1; (**c**) distribution of yield strength with respect to tensile test temperature; (**d**) ANN model training results.

Utilizing 150 tensile experimental sample data points from various heat treatment regimens and tensile test temperatures, a training set was constructed for the model. The relationship between partial characteristic parameters and yield strength for these samples is shown in Figure 7a–c. The dataset was divided into training and testing sets in an 8:2 ratio, following which the BP-ANN model was trained using the previously defined model parameters. The training outcomes are depicted in Figure 7d.

Model performance was assessed using *RMSE* and *MADP*, where the *RMSE* for the training set was 50.37, with an associated *MADP* of 3.77, and the testing set exhibited an *RMSE* of 50.13 with an associated *MADP* of 3.71. These results indicate a high level of accuracy and reliability relative to the model’s predictions. Furthermore, the similarity in *RMSE* and *MADP* values between the training and testing sets suggests that the model shows no significant signs of overfitting and possesses strong generalization capabilities.

### 4.3. Finite Element Simulation Results

**Figure 8:**
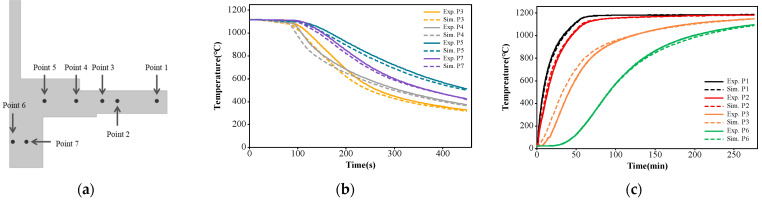
Finite element model calibration: (**a**) temperature measurement point locations; (**b**) temperature history curve during the heating process; (**c**) temperature history curve during the cooling process.

To ensure the accuracy of finite element simulation results, the thermal boundary conditions of the FEA model need to be calibrated before simulation. The specific method involves embedding several thermocouples in the turbine disk, referencing the measured temperature curves during the heat treatment heating and cooling process, and adjusting the model’s boundary conditions to match the simulated temperature curves with the measured ones at the corresponding thermocouple positions. The placement of the thermocouples is shown in Figure 8a. A comparison of the measured temperature history curve and the simulated temperature history curve is presented in Figure 8b,c, demonstrating a good alignment between the simulated and measured temperature curves.

**Figure 9:**
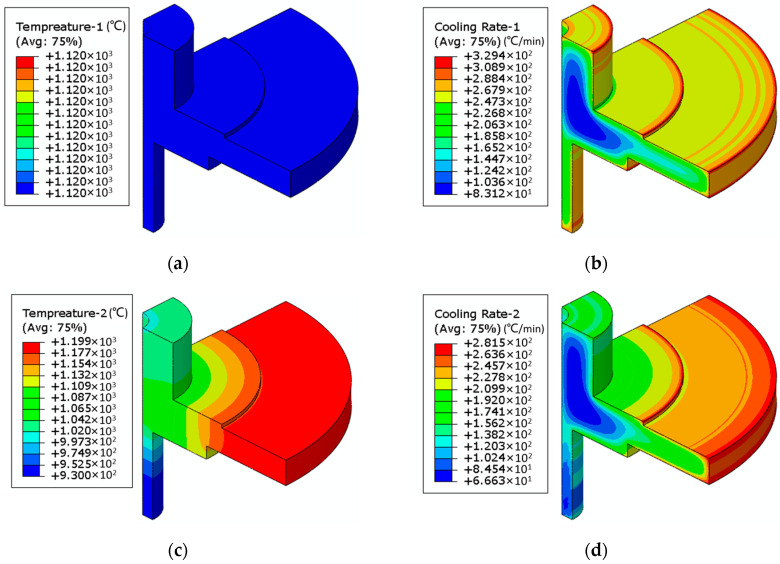
Finite element simulation results of DMHT: (**a**) subsolvus heat treatment temperature; (**b**) subsolvus heat treatment cooling rate; (**c**) supersolvus heat treatment temperature; (**d**) supersolvus heat treatment cooling rate.

In accordance with the thermal treatment specifications of DMHT, finite element simulations were conducted in two distinct steps. In the first step, a uniform furnace temperature of 1120 °C was applied for the subsolvus heat treatment. Subsequently, oil quenching was performed. The holding stage temperature distribution and oil quenching cooling rate are illustrated in Figure 9a,b.

In the second step of the heat treatment, a furnace temperature of 1200 °C was applied, with the hub region covered by insulation, resulting in a radial temperature gradient across the disk. This configuration effectively subjected the rim region to a full supersolvus heat treatment while the hub region underwent a subsolvus heat treatment. Similar to step one, oil quenching was performed after supersolvus temperature holding, and the associated temperature and cooling rate are depicted in Figure 9c,d.

The simulation results indicate that, during the second stage of the heat treatment process, the transitional region attained a heat treatment temperature of approximately 1150 °C, meeting the criteria of supersolvus heat treatment for the rim region and subsolvus heat treatment for the hub region. Furthermore, both stages of heat treatment exhibited similar distribution patterns of oil quench cooling rates, characterized by lower cooling rates in the central thick region of the hub.

### 4.4. Integration of FEA and BP-ANN

**Figure 10:**
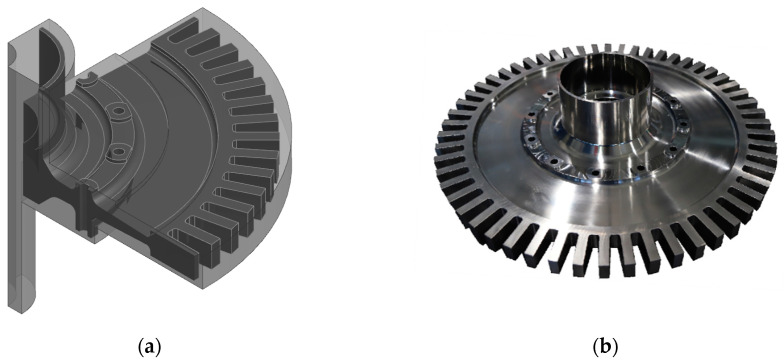
Heat-treated disk blank and the final shape of the disk: (**a**) 3D model of the disk; (**b**) photo of the disk.

The utilization of finite element analysis to obtain temperature and cooling rate distributions within the disk during the heat treatment process, with both parameters then used as inputs for the ANN model to predict disk tensile performance, represents an efficient method to assist in the design of the heat treatment scheme. However, in the manufacturing process of turbine disks, the heat treatment process is often conducted on the blank disk, which requires further machining. As a result, the simulation results of the heat treatment process for the DMHT need to be mapped onto the final shape turbine disk model. A schematic diagram illustrating the heat-treated disk blank and the final shape of the testing disk is shown in Figure 10.

Considering the distinctive characteristics of the disk component, which exhibits varying mechanical properties across different regions, traditional finite element simulations often rely on homogenized temperature-dependent and strain-rate-dependent material properties. However, these methods fail to represent accurately the variation of material properties on the workpiece. To address this issue, this study proposes a material property assignment approach. Based on the heat treatment information for each element of FEA, material properties were calculated using the ANN model and subsequently assigned to each finite element. This coupling of ANN and FEA allowed for a precise depiction of content mechanical performance distribution across the component. The integrated computational framework of ANN and FEA is depicted in Figure 1, with the ANN model and FEA results having been established in prior research.

The integrated low-cycle fatigue calculation process described in this study follows these steps:

(1) Integration of Trained ANN model: the trained ANN model was embedded into the finite element model using a user-defined subroutine USDFLD. This included scaling coefficients, the neural network weights, and biases for each layer. The inputs and output of the ANN model are defined as user-defined field variables.
(2) Finite element results mapping: the finite element prediction results obtained from the heat-treated blank model, such as HT temperatures and cooling rates, were mapped into the final shape turbine disk model as user defined fields.
(3) Material property mapping: the yield strength was linked to a user-defined field which was predicted by the ANN model.
(4) Structural analysis: calculations were carried out based on the disk ground testing conditions. Firstly, the thermal analysis was conducted according to the tested temperature results. Secondly, a rotational load was applied to the disk for the mechanical analysis.

This integrated approach provided a comprehensive analysis of the structural behavior by considering the influence of the heat treatment process on yield strength.

**Figure 11:**
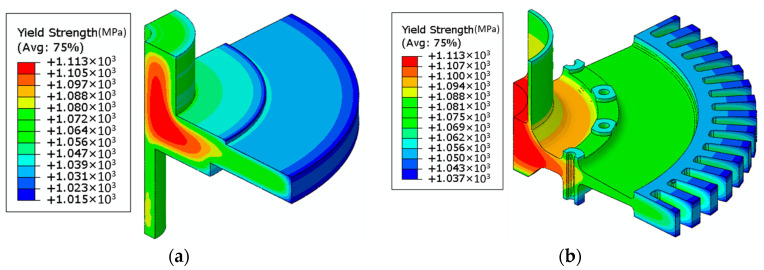
Tensile strength prediction at 550 °C: (**a**) yield strength of DMHT disk; (**b**) yield strength of the final shape of the turbine disk.

Before conducting the structural analysis, it is essential to validate the model’s accuracy. The model used 550 °C as the input parameter for tensile testing temperature, while the remaining input parameters were based on the mapping of the heat treatment results from the raw blank of the disk. The predicted tensile strength results of DMHT disk and the final shape of the turbine disk are shown in Figure 11.

**Figure 12:**
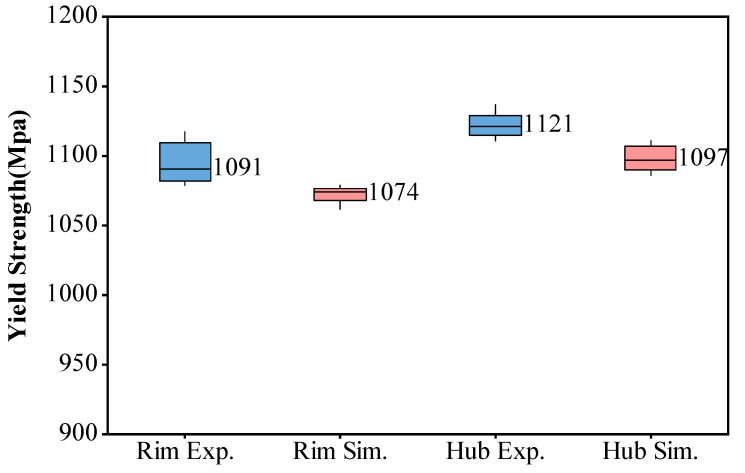
Yield strength prediction validation at 550 °C.

Tensile experiments were conducted at 550 °C on specimens taken from both the hub and rim regions of the final shape turbine disk, and the results were compared with the predicted data. As shown in Figure 12, at the test temperature of 550 °C, the predicted tensile strength in the hub region was 1097 MPa, while the median experimental result was 1121 MPa, indicating a slight underprediction of 24 MPa, an error of 2.1%. In the rim region, the predicted tensile strength was 1074 MPa, compared to the experimental median of 1091 MPa, resulting in a slight underprediction of 17 MPa, with an error of 1.6%. These results demonstrate that the overall trends in the experimental and predicted results were consistent. The errors in the predictions were rather small, indicating that the model’s performance was quite accurate and reliable for predicting the tensile strength.

**Figure 13:**
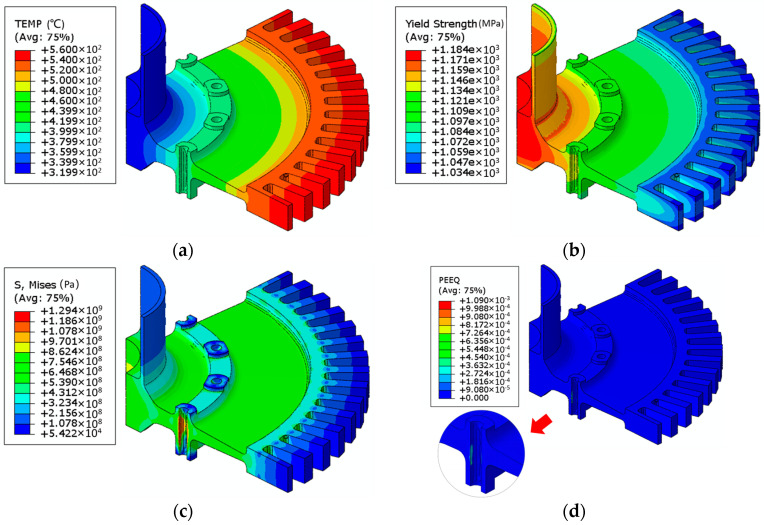
DMHT model for disk mapping and yield strength prediction: (**a**) temperature distribution during the test; (**b**) calculated material yield strength at the temperature of (**a**); (**c**) calculated stress distribution under testing conditions coupled with the material properties from (**b**); (**d**) calculated equivalent plastic strain distribution under testing conditions coupled with the material properties from (**b**).

The integrated simulation of FEA and ANN was conducted under low-cycle fatigue experiment conditions of the turbine disk. The turbine disk operated at an actual speed of 15,000 rad/min, with measured temperature distributions of 320 °C at the disk hub and 570 °C at the rim. Temperature distribution and corresponding predicted tensile strength are displayed in Figure 13a,b. The finite element results calculated using the integrated model can effectively capture the uneven yield strength caused by the non-uniform temperature distribution after loading, enhancing the accuracy of subsequent stress calculations. The calculated Von Mises stress results are presented in Figure 13c, and the equivalent plastic strain results are shown in Figure 13d; minor yielding can occur, possibly, at the bolt–hole locations. This approach enabled the prediction of the yield strength distribution under actual working conditions of the turbine disk and the identification of failure regions, providing an efficient means for turbine disk heat treatment design. Additionally, by using the predicted yield strength distribution and stress state under actual working conditions, it is possible to perform topological optimization of the turbine disk geometry to reduce disk weight and enhance material utilization.

## 5. Discussion

### 5.1. Qualitative Analysis of the Strengthening Mechanism

The strengthening mechanism in powder metallurgy (P/M) nickel-based superalloys primarily involves solid solution strengthening, grain boundary strengthening, and precipitation strengthening and can be summarized as follows:

(11) $$\sigma_{ys}=\sigma_{ss}+\sigma_{gs}+\sigma_{ps}$$

For a specific alloy, at equilibrium state, the volume fractions of various alloy phases are temperature-dependent and remain unchanged regardless of variations in heat treatment processes. Consequently, for the F4113A alloy, the contribution of solid solution strengthening to yield strength remains constant. However, grain boundary strengthening and precipitation strengthening are highly correlated with the morphological characteristics of the alloy microstructure. Moreover, under different heat treatment regimes, the alloy microstructure exhibits significant variations, thereby becoming the primary factor influencing whether the alloy exhibits excellent tensile property or not.

**Figure 14:**
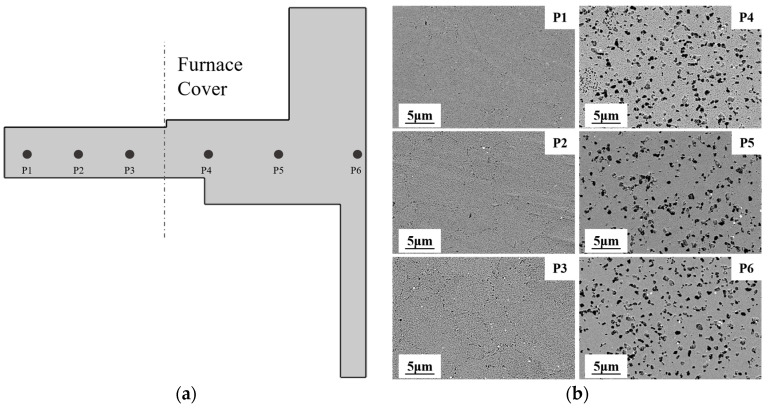
SEM micrographs of FGH4113A dual-performance turbine disk: (**a**) sampling point of turbine disk; (**b**) low magnification showing the γ′ size in different regions of the turbine disk.

To validate the relationship between microstructure and yield strength, samples were collected from different regions of the disk component for microscopic observation of their microstructures in Figure 14, including the measurement of the average grain size and average size of the secondary γ′ phase.

#### 5.1.1. Solid Solution Strengthening

Solid solution strengthening in the γ and γ′ phases has been commonly considered for the calculation of the overall yield strength. The chemical composition of each phase directly affects the extent of solid solution strengthening [29,30]. The contribution of solid solution strengthening to the γ and γ′ phase, ${\sigma_{sss}}^{\gamma }$ and ${\sigma_{sss}}^{\gamma^{\prime }}$, can be written as:

(12) $${\sigma_{sss}}^{\gamma }=\left(1-f_{\gamma^{\prime }}\right){\left[\sum_{i}{\left(S_{i}^{\gamma }\right)}^{2}\right]}^{1/2}$$

(13) $${\sigma_{sss}}^{\gamma^{\prime }}=f_{\gamma^{\prime }}\sum_{i}{\left(S_{i}^{\gamma^{\prime }}\right)}^{2}$$

Here, $f_{\gamma^{\prime }}$ is the volume fraction of the γ′ phase, $S_{i}^{\gamma }$ and $S_{i}^{\gamma^{\prime }}$ are the degree of solid solution strengthening in the γ and γ′ phases, respectively, and can be explained as:

(14) $${S_{i}}^{\gamma }={\beta_{i}}^{\gamma }{x_{i}}^{\gamma }$$

(15) $${S_{i}}^{\gamma^{\prime }}={\beta_{i}}^{\gamma^{\prime }}{\left({x_{i}}^{\gamma^{\prime }}\right)}^{1/2}$$

The ${x_{i}}^{\gamma }$ and ${x_{i}}^{\gamma^{\prime }}$ are determined by the concentration of element i in each precipitate phase, ${\beta_{i}}^{\gamma }$ and ${\beta_{i}}^{\gamma^{\prime }}$ are constants related to the atomic size and modulus.

#### 5.1.2. Grain-Boundary Strengthening

**Figure 15:**
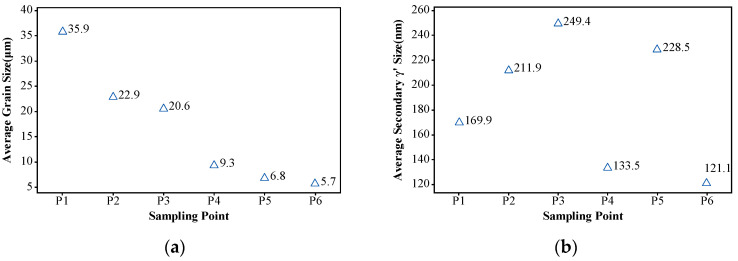
Statistics of the microstructure in dual-performance turbine disk: (**a**) the average grain size; (**b**) the secondary γ′ size.

The contribution of grain boundary strengthening to the room temperature yield strength in the FGH4113A alloy is commonly represented using the Hall–Petch relationship, expressed as follows:

(16) $$∆\sigma_{gs}=fk_{y}{d_{m}}^{-1/2}$$

where f is the volume fraction of the γ grain, $k_{y}$ represents the Hall–Petch coefficient, and $d_{m}$ denotes the average grain size. According to the Hall–Petch relationship, the contribution of grain boundary strengthening to the yield strength increases as the grain size decreases. From Figure 15a, it is evident that, during the DMHT process, the insulation-covered area of the turbine disk maintained a smaller grain size. This, effectively, enhances the contribution of grain boundary strengthening to the yield strength of the turbine disk hub.

At high temperatures, thermal activation promotes the movement of dislocations, resulting in the activation of a greater number of slip systems compared to room temperature. Relative to the matrix, the strength of grain boundaries diminishes rapidly with increasing temperature. Under the influence of applied stress, this manifests as extensive intergranular slip, where grain boundaries lose their ability to impede dislocation motion. Concurrently, at high temperatures, grain boundaries are prone to diffusion, causing their migration, further exacerbating deformation. Consequently, grain boundary strengthening diminishes and can even vanish as temperature rises.

#### 5.1.3. Precipitation Strengthening

The precipitation strengthening mechanisms primarily include weak pair coupling, strong pair coupling, and Orowan bowing mechanisms. The interaction between dislocations and the γ′ phase is significantly influenced by the size of the γ′ phase, and the enhancement of the strengthening is attributed to the formation of antiphase boundaries. When the size of the γ′ phase is approximately smaller than 20 nm, multiple γ′ phase particles might exist within two adjacent dislocations, a condition known as weak-pair coupling [4]. If a following dislocation enters the γ′ phase while the trailing dislocation is still within the γ′ phase, it is termed as strong-pair coupling [4]. The strengthening effect of the weak-pair coupling mechanism increases with a decrease in the size of the γ′ phase, whereas the strong-pair mechanism exhibits the opposite behavior. As the size of the γ′ phase further increases and interparticle spacing enlarges, the force required for dislocation bowing diminishes, gradually shifting the interaction mechanism with the γ′ phase toward the predominant Orowan bowing mechanism.

Formulas for weak-pair coupling and strong-pair coupling are as follows [11]:

(17) $$\Delta \tau_{weak}=\frac{1}{2}{\left(\frac{\gamma_{APB}}{b}\right)}^{\frac{3}{2}}{\left(\frac{bd_{s}f}{T}\right)}^{\frac{1}{2}}A-\frac{1}{2}\left(\frac{\gamma_{APB}}{b}\right)f_{\gamma^{\prime }}$$

(18) $$\Delta \tau_{strong}=\frac{1}{2}\left(\frac{Gb}{d_{s}}\right){f_{\gamma^{\prime }}}^{1/2}0.72\omega {\left(\frac{\pi d_{s}\gamma_{APB}}{\omega Gb^{2}}-1\right)}^{1/2}$$

Among these, τ represents the critical shear stress, $\gamma_{APB}$ stands for the anti-phase boundary energy per unit area, b denotes the Burgers vector, $d_{s}$ signifies the mean diameter of the γ′ phase, $f_{\gamma^{\prime }}$ represents the volume fraction of the γ′ phase, A is the geometric factor, G denotes the shear modulus of the γ′ phase, ω is a parameter reflecting the elastic and it assumes a value of 1, while T is a line tensor, expressed as follows:

(19) $$T=\frac{Gb^{2}}{2}$$

According to the formula, it is evident that the most influential variables affecting strengthening are the size and volume fraction of the γ′ phase. When the γ′ size meets the critical dimension, it activates both the weak-pair coupling and strong-pair coupling cutting mechanisms.

If the size of the γ′ phase is large, the interaction mechanism between dislocation pairs and the γ′ phase will transition from the strong-pair coupling mechanism to the Orowan bowing mechanism, satisfying the following formula:

(20) $$\tau_{oro}=\frac{GB}{L}$$

where *L* represents the effective interparticle spacing:

(21) $$L=\sqrt{\frac{8}{3\pi f}}d_{s}-d_{s}$$

**Figure 16:**
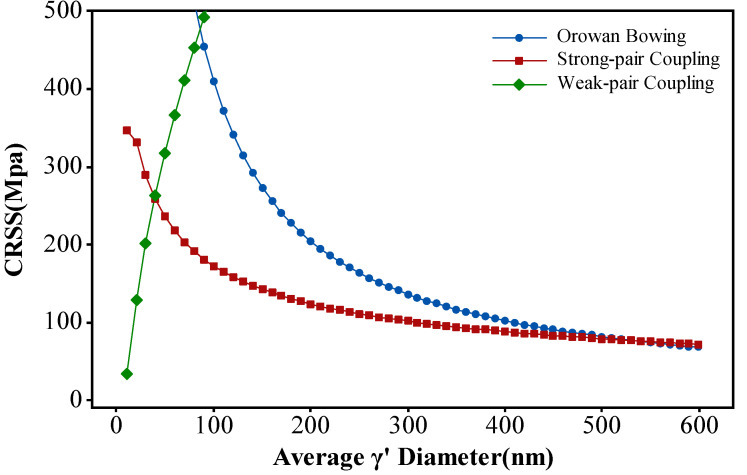
The critical shear stress required for the γ′ phase size and the corresponding dislocation interaction mechanisms in the FGH4113A alloy.

At the equilibrium state, the volume fraction of the γ′ phase is primarily temperature-dependent. When its size is larger, resulting in lower density, the interparticle spacing increases. Consequently, the force required for dislocation bypass around the γ′ phase decreases. Calculations from the formula indicate that the critical size for weak coupling and strong coupling of the γ′ phase is approximately 40 nm, while the critical size for strong coupling and Orowan bowing mechanism of the γ′ phase is around 530 nm, as shown in Figure 16. When dislocations come into contact with the γ′ phase, the precipitation strengthening mechanism, requiring a lower critical shear stress, is preferably initiated. Hence, when the gamma phase is below 530 nm, the precipitation strengthening mechanism is dominated by the coupling mechanism. Conversely, the Orowan bowing mechanism predominantly operates in precipitation strengthening when the gamma phase exceeds this threshold.

During the DMHT process of the turbine disk, the temperature in the hub region remains lower than the alloy’s solidus temperature, resulting in incomplete dissolution of the primary γ′ phases into the γ matrix. As depicted in Figure 14b, numerous remnants of the primary γ′ phases persist in the hub region. In contrast, the rim region exhibits the opposite behavior, as the heat treatment temperature exceeds the alloy’s solidus temperature, resulting in the complete dissolution of γ′ into the γ matrix.

Combining the analysis of the cooling rates, in the rim region, the heat treatment cooling rate gradually decreases radially from the center toward the edge. Consequently, the size of the precipitated γ′ phases increases progressively during the cooling process. In the hub region, due to the central hole penetrating through the disk, the cooling rate in the central part, in the radial direction, is the slowest, while both the outer and inner regions exhibit faster cooling rates. This results in the largest size of the γ′ phases in the central part, while the corresponding sizes in the outer and inner regions are smaller than those in the central part. Simultaneously, although the cooling rate in the hub region is slower than that in the edge region, the hub’s heat treatment temperature is actually sub-solidus, allowing the undissolved primary γ′ phases to occupy the elements necessary for the formation of the γ′ phases. Consequently, the secondary γ′ phases are smaller in the hub region compared to the rim.

From Figure 14b, it is evident that, in the rim region of the turbine disk, the γ′ phases fully dissolved after the heat treatment and reprecipitated as smaller-sized secondary γ′ phases. Meanwhile, in the hub region of the turbine disk, both the undissolved larger-sized primary γ′ phases and the reprecipitated secondary γ′ phases coexist. Additionally, as depicted in Figure 15b, in the rim region of the turbine disk, the precipitation strengthening mechanism is predominantly governed by the dislocation coupling mechanism, whereas, in the hub region, both dislocation cutting and Orowan bowing mechanisms act concurrently. Compared to the Orowan bowing mechanism, the dislocation coupling mechanism contributes more significantly to the yield strength, particularly in the hub region where the smaller grain size amplifies the effect of grain boundary strengthening. Hence, a combination of various strengthening mechanisms results in the difference in yield strength between the hub and rim regions.

### 5.2. Validity of the Integrated Model

This study establishes an ANN model to predict the yield strength of the turbine disk based on a dataset of tensile experimental data. The training results of the model can accurately reflect the trend of the yield strength under different heat treatment parameters, but there is still a certain error in the specific numerical value. There are several sources of error. First, in the process of establishing the model, the influence of the initial microstructure on tensile properties was not considered. In the process of material preparation, due to process limitations, both HIP and HEX processes will bring a certain degree of microstructure heterogeneity, which is one of the sources of error. Second, in the cooling process after the heat treatment, cooling is not a linear process and strengthening phases precipitate and grow faster at high temperatures. Using the average cooling rate as an input to the ANN model cannot accurately reflect the trend of material microstructure changes with decreasing temperature, which will also affect the final prediction results. Third, in the process of DMHT simulation, due to the complexity of actual working conditions, the simulation results and actual results cannot fully coincide, which also causes errors.

To address the aforementioned issues, during the training process of the ANN model, more factors affecting the tensile strength due to the heat treatment need to be considered as input parameters for the model, or the heat treatment parameters need to be refined and the amount of training set data need to be increased. In the FEA simulation of the heat treatment process, more temperature measurement points need to be added to the disk for accurate temperature measurement to calibrate the model. However, this also greatly increases the difficulty and complexity of data acquisition. 

### 5.3. Application in Turbine Disk Design

If there is a sufficient amount of material data available, the integrated computational approach described in this study could be applicable to construct other material property models, such as creep and fatigue models, and can be used for the rapid design of dual-performance turbine disks. At the rim, fatigue life can be improved by coarse grain, which is obtained from the supersolvus heat treatment. At the hub, sub-solution heat treatment can keep the fine grain size, which is beneficial to the creep performance. By optimizing processes and the structural design, it is possible to make better use of materials at different locations, achieving a more even service life and, consequently, reducing component weight.

In the case of the need to optimize the heat treatment process for the DMHT disk, various plans involving the disk and insulation geometry and DMHT parameters can be designed for the integrated calculation. The feasibility of these plans is assessed through the calculation results and adjusted according to the property requirement of the turbine disk. For example, as described in this study, there is a stress concentration issue at the bolt–hole positions during loading, as shown in Figure 13d. However, the DMHT disk does not exhibit the highest yield strength at the bolt–hole positions, as indicated in Figure 11a. An analysis has revealed that the bolt–hole positions on the original DMHT disk are in a transitional zone between supersolvus and subsolvus heat treatment, while the supersolvus region has a higher yield strength. Therefore, one of the approaches to address this issue could be increasing the coverage area of the insulation to fully cover the heat treatment bolt–hole positions.

In the structural design of the turbine disk, calculations can be performed based on the given geometry of the turbine disk, the heat treatment process, and the loading conditions. Subsequently, structural optimizations of the turbine disk can be carried out according to the simulation results. In regions where stress concentrations occur, the original design can be retained or reinforced. In areas where there is no stress concentration, materials can be reduced to create a more structurally efficient turbine disk.

## 6. Conclusions

The yield strength prediction of the dual-performance turbine disk was developed using a method of integration of FEA and ANN. The following conclusions can be drawn from this study:

(1) An ANN model for predicting the yield strength of the turbine disks based on the DMHT parameters was constructed using tensile test samples from medium-sized experimental disks. RMSE and MAPD for the training set were 50.37 and 3.77, respectively, while, for the testing set, they were 50.13 and 3.71, respectively. The model exhibited good predictive performance.
(2) Through FEA, the DMHT process was simulated, and the temperature and cooling rate distribution during the DMHT process of the disk were obtained. Prior to the simulation, the thermal boundary conditions were calibrated, ensuring that the simulation results were highly reliable.
(3) A coupled method of FEA and ANN was established for yield strength prediction. The temperature and cooling rate data from the DMHT simulation results were used as input parameters for this coupled method, which successfully predicted the yield strength of the DMHT model.
(4) An integrated model of FEA and ANN was established using a user subroutine. In the loading process of the turbine disk, the yield strength was predicted based on temperature calculation results, and material properties were updated automatically. This approach successfully predicted the stress and strain states of the turbine disk during the actual loading process.

## Figures and Tables

**Table 1:** Chemical composition of FGH4113A alloy (wt.%).

Co	Cr	Al	Ti	W	Mo	Ta	Nb	Hf	Ni
19.0	13.0	3.0	3.7	4.0	4.0	1.0	1.2	0.2	etc.

## Data Availability

The original contributions presented in the study are included in the article, further inquiries can be directed to the corresponding authors.

## References

[1] Thellaputta G.R., Chandra P.S., Rao C.S.P. (2017). Machinability of Nickel Based Superalloys: A Review. Mater. Today Proc..

[2] Nowotnik A., Kubiak K., Sieniawski J., Rokicki P., Pędrak P., Mrówka-Nowotnik G. (2014). Development of Nickel Based Superalloys for Advanced Turbine Engines. MSF.

[3] Wang Y.L., Li Y., Zhang H., Guo J.Z. (2021). Hot Deformation Induced Microstructure Evolution of a Novel As-Extruded Ni-Based P/M Superalloy. Philos. Mag..

[4] Kozar R.W., Suzuki A., Milligan W.W., Schirra J.J., Savage M.F., Pollock T.M. (2009). Strengthening Mechanisms in Polycrystalline Multimodal Nickel-Base Superalloys. Metall. Mater. Trans. A.

[5] Wu Y., Li C., Xia X., Liang H., Qi Q., Liu Y. (2021). Precipitate Coarsening and Its Effects on the Hot Deformation Behavior of the Recently Developed γ′-Strengthened Superalloys. J. Mater. Sci. Technol..

[6] He D.-G., Lin Y.C., Jiang X.-Y., Yin L.-X., Wang L.-H., Wu Q. (2018). Dissolution Mechanisms and Kinetics of δ Phase in an Aged Ni-Based Superalloy in Hot Deformation Process. Mater. Des..

[7] Zhang X., Chen Y., Cao L., Sun Y., Li J., Cheng X., Tian G. (2023). Microstructures and Tensile Properties of a Grain-Size Gradient Nickel-Based Superalloy. J. Alloys Compd..

[8] Tian G., Jia C., Liu J., Hu B. (2009). Experimental and Simulation on the Grain Growth of P/M Nickel-Base Superalloy during the Heat Treatment Process. Mater. Des..

[9] Lemsky J. (2004). Assessment of NASA Dual Microstructure Heat Treatment Method for Multiple Forging Batch Heat Treatment.

[10] Lemsky J. (2005). Assessment of NASA Dual Microstructure Heat Treatment Method Utilizing Ladish SuperCooler^TM^ Cooling Technology.

[11] Collins D.M., Stone H.J. (2014). A Modelling Approach to Yield Strength Optimisation in a Nickel-Base Superalloy. Int. J. Plast..

[12] Li W., Ma J., Kou H., Shao J., Zhang X., Deng Y., Tao Y., Fang D. (2019). Modeling the Effect of Temperature on the Yield Strength of Precipitation Strengthening Ni-Base Superalloys. Int. J. Plast..

[13] Gabb T.P., Kantzos P.T., Telesman J., Gayda J., Sudbrack C.K., Palsa B. (2011). Fatigue Resistance of the Grain Size Transition Zone in a Dual Microstructure Superalloy Disk. Int. J. Fatigue.

[14] Jiang R., Wang Y.C., Zhang L.C., Chen Y., Zhang H., Wang Z.B., Song Y.D. (2023). Fatigue Crack Propagation Behavior of the Grain Size Transition Zone in a Dual Microstructure Turbine Disc. Int. J. Fatigue.

[15] Wu L., Osada T., Watanabe I., Yokokawa T., Kobayashi T., Kawagishi K. (2021). Strength Prediction of Ni-Base Disc Superalloys: Modified Γ’ Hardening Models Applicable to Commercial Alloys. Mater. Sci. Eng. A.

[16] Cao C. (2023). Prediction of Concrete Porosity Using Machine Learning. Results Eng..

[17] Fatriansyah J.F., Suhariadi I., Fauziyyah H.A., Syukran I.R., Hartoyo F., Dhaneswara D., Lockman Z., Fauzi A., Rohman M.S. (2023). Prediction and Optimization of Mechanical Properties of Ni Based and Fe–Ni Based Super Alloys via Neural Network Approach with Alloying Composition Parameter. J. Mater. Res. Technol..

[18] Zhu Y., Cao Y., He Q., Luo R., Zhang J., Di H., Huang G., Liu Q., Xiao J. (2022). Machine Learning Neural-Network Identification for Dynamic Recrystallization Grains during Hot Deformation of Nickel-Based Superalloy. Mater. Charact..

[19] Zhang C., Li Y., Jiang B., Wang R., Liu Y., Jia L. (2022). Mechanical Properties Prediction of Composite Laminate with FEA and Machine Learning Coupled Method. Compos. Struct..

[20] Lin Y.C., Nong F.-Q., Chen X.-M., Chen D.-D., Chen M.-S. (2017). Microstructural Evolution and Constitutive Models to Predict Hot Deformation Behaviors of a Nickel-Based Superalloy. Vacuum.

[21] Wen H., Jin J., Tang X., Wang X., Yang H., Zhang Y., Zhang M., Deng L., Wei Q., Chen J. (2023). Machine Learning-Assisted Constitutive Modeling of a Novel Powder Metallurgy Superalloy. Int. J. Mech. Sci..

[22] Gao J., Tong Y., Zhang H., Zhu L., Hu Q., Hu J., Zhang S. (2023). Machine Learning Assisted Design of Ni-Based Superalloys with Excellent High-Temperature Performance. Mater. Charact..

[23] Liu Z., Wang C., Cheng J., Guo J. (2023). An Improved Grain Growth Model and Its Application in Gradient Heat Treatment of Aero-Engine Turbine Discs. Materials.

[24] Huang G., Liu G.Q., Feng M., Zhang M., Hu B., Wang H. (2018). The Effect of Cooling Rates from Temperatures above the Γ’ Solvus on the Microstructure of a New Nickel-Based Powder Metallurgy Superalloy. J. Alloys Compd..

[25] Semiatin S.L., Mahaffey D.W., Levkulich N.C., Senkov O.N., Tiley J.S. (2018). The Effect of Cooling Rate on High-Temperature Precipitation in a Powder-Metallurgy, Gamma/Gamma-Prime Nickel-Base Superalloy. Metall. Mater. Trans. A.

[26] Wu H., Huang Z., Zhou N., Chen J., Zhou P., Jiang L. (2019). A Study of Solution Cooling Rate on γ′ Precipitate and Hardness of a Polycrystalline Ni-Based Superalloy Using a High-Throughput Methodology. Mater. Sci. Eng. A.

[27] Zhu L., Pan H., Cheng J., Xiao L., Guo J., Ji H. (2022). Dendrite Evolution and Quantitative Characterization of Γ’ Precipitates in a Powder Metallurgy Ni-Based Superalloy by Different Cooling Rates. J. Alloys Compd..

[28] Yang W.P., Liu G.Q., Wu K., Hu B.F. (2014). Influence of Sub-Solvus Solution Heat Treatment on Γ’ Morphological Instability in a New Ni–Cr–Co-Based Powder Metallurgy Superalloy. J. Alloys Compd..

[29] Goodfellow A.J., Galindo-Nava E.I., Schwalbe C., Stone H.J. (2019). The Role of Composition on the Extent of Individual Strengthening Mechanisms in Polycrystalline Ni-Based Superalloys. Mater. Des..

[30] Goodfellow A.J., Galindo-Nava E.I., Christofidou K.A., Jones N.G., Boyer C.D., Martin T.L., Bagot P.A.J., Hardy M.C., Stone H.J. (2018). The Effect of Phase Chemistry on the Extent of Strengthening Mechanisms in Model Ni-Cr-Al-Ti-Mo Based Superalloys. Acta Mater..

